# Patient representatives: Crucial members of health‐care working groups facing an uncertain role and conflicting expectations. A qualitative study

**DOI:** 10.1111/hex.13249

**Published:** 2021-05-05

**Authors:** Anna Hult, Ewa Lundgren, Eva Jangland

**Affiliations:** ^1^ Department of Surgical Sciences Uppsala University Uppsala Sweden

**Keywords:** cancer, group processes, health‐care working groups, patient and public involvement, patient participation, patient representatives, qualitative research, Sweden

## Abstract

**Background:**

Patient representatives (PRs) have been involved for decades in health‐care development, and their participation is increasingly sought in health‐care working groups (HCWGs) on every level. However, information on how the role could be further developed and teamwork improved remains sparse.

**Objective:**

To explore the role of patient representatives in clinical practice guideline (CPG) monitoring groups, to describe their contributions and identify possibilities of improvement.

**Design:**

Qualitative design using semi‐structured interviews analysed by content analysis.

**Setting and participants:**

Interviews were conducted with 11 PRs, 13 registered nurses, and 9 physicians, all members of national committees monitoring CPGs for cancer in Sweden.

**Results:**

Most participants considered the PR role important but mentioned several problems. PRs’ contributions were hampered by uncertainties about their role, the low expectations of other group members and their sense that their contributions were often disregarded. Some professionals questioned whether PRs were truly representative and said some topics could not be discussed with PRs present.

**Conclusion:**

This study highlights the fundamental problems that remain to be solved despite the long involvement of PRs in HCWGs. Even though the PR role and teamwork differed between the groups, most PRs need to be empowered to be actively involved in the teamwork and have their engagement and knowledge fully utilized. Enhancing teamwork through clarifying roles and expectations could lead to more inclusive and equal teams able to work more effectively towards the goal of improving health care.

**Patient or public contribution:**

PRs were information givers in data collection.

## 1 INTRODUCTION

Patient and public involvement (PPI), increasingly recognized in planning and improving health care and now central to health reform agendas in the Western world,^1^, ^2^, ^3^ continues to lack a consistent definition.^1^, ^2^ Consultation, engagement, participation, partnership and co‐production are all described as aspects of PPI, implying greater or lesser levels of involvement.^1^ PPI can vary from participation in public polls, surveys, seminars, workshops, focus groups or individual interviews to service in development or monitoring groups for clinical practice guidelines (CPGs).^4^ Best practices for PPI are also unclear,^1^ and professionals sometimes resist patient participation.^4^

Patient participation has been defined in several ways.^5^, ^6^ This study is theoretically based on the fundamentals of care framework,^7^ with a specific focus on patient participation and the view that the patient is a resourceful individual who receives, comprehends and possesses information and knowledge that should be shared and respected.^5^ Patient participation can occur on the direct care (micro), health‐care organization (meso), and societal and governmental (macro) levels.^8^, ^9^ The fundamentals of care highlights a holistic perspective on the patient and address the critical need to embed the patient's voice at many levels of the health‐care system.^7^, ^10^

As patients’ experiences and general views on health care are increasingly required, patient representatives (PRs) are often mandatory in local and national health‐care working groups (HCWGs). Organizations with PPI experience have reported that participants in CPG working groups require certain abilities or skills, such as communication and teamwork, for group processes to be effective.^4^ Professionals sometimes question the added value of patients’ input, considering themselves already familiar with most patient findings.^11^ However, patient involvement is reported to make practice‐based knowledge more explicit in guidelines and to contribute to patient‐relevant topics receiving greater priority.^11^, ^12^ PRs are also reported to influence the implementation and dissemination of CPGs^12^; however, the goals of involving PRs are often implicit or vaguely articulated, making it difficult to estimate their impact.^13^ Wheelan describes a working group as members striving to develop an efficient and effective structure to accomplish shared goals.^14^ According to the integrated model of group development, a working group does not become a functioning team until the goals are established and methods to accomplish them are in place.^14^

Involving PRs throughout the process of guideline development was evaluated as positive, but integrating patients’ perspectives with research evidence was described as challenging.^11^ Whether PRs can or should represent a broad patient constituency or whether they should bring their own personal experience to the team remain topics of discussion and are key recruitment questions.^1^, ^4^, ^11^, ^13^ Difficulties in recruiting and supporting PRs and PRs’ lack of familiarity with scientific and medical terminology are also described as barriers to their work in HCWGs; clear expectations, training, support, and involving more than a single patient have all been described as facilitators.^4^

An official government report, *A National Cancer Strategy for the Future*,^15^ resulted in the establishment of regional cancer centres (RCCs) in each of the six health‐care regions in Sweden in 2011. The RCCs focus on patients with cancer, aiming to increase health‐care quality, results and equality.^16^ In line with this purpose, RCCs support the 49 (2020) national CPGs for the care of cancer patients. The care programmes include recommendations, quality indicators and target levels and are updated annually by a monitoring group. All these groups include at least one PR (or relative) along with the professionals. All RCCs provide PRs with support and education.

Although PRs have been involved in developing health care for decades, and their participation is increasingly sought on all levels, the impact of their contributions is difficult to measure and seems to vary widely.^2^, ^9^, ^12^, ^17^ PRs’ participation in HCWGs is also still hampered by obstacles,^1^, ^4^ and reports on how to further develop the PR role remain sparse. Since PRs are increasingly expected in health‐care working groups on all levels in Sweden, it is important to deepen the understanding of their role and contributions to the groups and possibilities for improvements. To the best of our knowledge, the PR role in CPG monitoring groups, an example of HCWGs on national level, has not previously been studied in Sweden.

The aim of the study was to explore the role of patient representatives in national clinical practice guidelines monitoring groups, to describe their contributions and to identify possibilities for improvement.

## 2 METHODS

### 2.1 Design

The study had a qualitative design using semi‐structured interviews with members of national CPG‐monitoring groups for cancer diseases.

### 2.2 Participants and setting

Convenience sampling was used to recruit members of Swedish national CPG‐monitoring groups for cancer. From approximately 40 CPG‐monitoring groups responsible for cancers with different incidences and managed by different medical specialties, a total of seven were included, with the possibility to add more CPGs if needed to reach saturation (Table 1). Mailing lists with the names and addresses of members were provided by the RCCs. These groups included PRs (patients and relatives) and clinicians, mainly registered nurses (RNs) and physicians. All participants in the study were permanent members of a CPG‐monitoring group, and the PRs were invited to participate in the same way as the professionals.

**TABLE 1:** Characteristics of participants

Specific cancer	Number of participants			
	Physicians	Registered nurses	Patient representatives	Total
Prostate	2	3	2	7
Ovarian	1	1	1	3
Blood^a^	2	1	1	4
Thyroid gland	2	3	2	7
Lung	0	2	2	4
Anus	1	1	1^b^	3
Brain	1	2	2^b^	5
Total	9	13	11	33
Women/men	5/4	12/1	7/4	
Age in years (mean)	39‐64 (52)	31‐60 (47)	33‐81 (61)	

Note

Physicians and nurses participated as experts in cancer care. Patients and relatives represented patients suffering from the selected cancer.

^a^ Aggressive B‐cell or T‐cell lymphoma.

^b^ Relatives only.

All nurses and PRs on the lists were approached via e‐mail with information about the study and a request for an interview. Physicians outnumbered the other two categories, so a letter was sent to every third physician in each group, excluding the group chairperson. The sole exception to this approach was made for physicians on the CPG‐monitoring group for lung cancer, who were all eventually invited. Despite supplementary information given to the chairperson, the 14 physicians in this group jointly decided not to take part in the study and offered no explanation. All individuals were reminded twice by e‐mail. All the physicians and nurses in the monitoring groups were experienced and highly qualified in their medical fields. University hospitals outnumbered county hospitals. Of 11 PRs, 8 were members of patient associations. In addition to the physicians in the group for lung cancer, four physicians and three nurses actively declined to take part in the study, and nine and eight, respectively, did not respond to repeated requests. All patients and relatives approached agreed to take part in the study.

### 2.3 Data collection

Two of the authors (AH and EL) conducted the interviews from August 2017 to May 2018. All three authors had worked with patients with cancer, either as RNs on surgical wards (AK and EJ) or as an MD specialized in endocrine surgery and breast cancer (EL), but none had ever worked with PRs on health‐care teams. Each interviewer initially conducted one pilot interview and the technique was modified after thorough discussion with the third author, who is experienced in qualitative analysis. Interviewers met with 11 PRs, 9 physicians and 13 RNs. Details and characteristics of participants are presented in Table 1.

The participants were from various locations in Sweden. All interviews, but one, were conducted by telephone. One interview was conducted face‐to‐face with an RN who the interviewer was able to visit at her workplace. All interviews were recorded and lasted for approximately 20‐30 minutes. The interview questions are shown in Table 2. Probing questions were used to encourage participants to clarify and expand upon their responses.

**TABLE 2:** Interview questions

Patient representatives (PRs)	Professionals
Would you please describe your view of the role of PRs in CPG development groups?	Would you please describe your view of the role of PRs in CPG development groups?
Was there any change in your role over time?	Was there any change in PR’s role over time?
Are there expectations about how you should prepare before the meetings? If yes, what are they?	Are there expectations about how the PRs should prepare before the meetings? If yes, what are they?
Are you responsible for any part of the guideline?	Are PRs responsible for any part of the guideline?
Do you have any support?	Do the PRs have any support?
How were you introduced to the group?	How were the PRs introduced to the group?
Have you been part of the group from the start? (Asked only of PRs)	
Give an example of when your opinion influenced (possibly changed) the guidelines.	Give an example of when a PR’s opinion influenced (possibly changed) the guidelines.
Give an example of when you suggested a change, but the proposal was turned down.	Give an example of when the PR suggested a change, but the proposal was turned down.
Give examples of the advantages of having a PR in the group.	Give examples of the advantages of having a PR in the group.
Give examples of difficulties associated with having a PR in the group.	Give examples of difficulties associated with having a PR in the group.
How could the role of PRs develop in the future?	How could the role of PRs develop in the future?

The interviews were transcribed word for word by a secretary. The two authors responsible for the interviews listened again to most of the interviews before confirming the transcripts. Participants were included until data were considered saturated.^18^

### 2.4 Data analysis

The material was analysed using conventional content analysis,^19^ an inductive approach without pre‐conceived categories. The analytic process is presented in Tables 3 and 4. Throughout the analysis, the authors reflected upon whether their backgrounds and experiences might risk biasing the results.

**TABLE 3:** Analytic process

The transcripts were read by each author independently to achieve immersion and obtain a sense of the whole.
All authors separately coded a number of interviews to identify and sort meaning units for preliminary coding by highlighting the words from the text that appeared to capture key thoughts and concepts. During this part of the analysis, all authors came together in a face‐to‐face meeting and discussed the preliminary coding until they came to agreement.
The first author led the analysis and coded the rest of the interviews. This step was performed in close collaboration with the second author. The codes were sorted into preliminary sub‐categories (clusters) based on how different codes were related and linked. The labelling of the sub‐categories, and the content was thoroughly discussed. The sub‐categories were thereafter organized into categories. Based on the coding scheme, the content of the categories was developed. This step included discussion of similarities and differences in the PR role in different monitoring groups. It also included a discussion of the relation between the categories.
All interviews were re‐read by the first author, and the findings were compared with the original transcriptions to ensure that they reflected the views of participants in different roles and different monitoring groups.
The rigour of the analysis was established through this detailed description of the steps and examples of the process shown in Table 4. Quotations from the interviews were added to confirm the content of the categories. This process enhances the credibility of the analysis, as the last author (experienced in qualitative research) was involved in debriefing sessions including reading and reviewing transcripts, coding and identifying emerging categories.^33^ The findings were thoroughly discussed among all authors until agreement was obtained for each category.

**TABLE 4:** Examples of analysis, from meaning unit to category

Meaning unit	Code	Category
For one thing, I think it's extremely important that patients are present, because that affects the tone of the discussion, the atmosphere, and the focus, and all that, you know. (PR)	Physical presence affects the focus and atmosphere of the discussions	Patient representatives’ participation in the group: a demanding role with different expectations from the professionals
In the group everything's so extremely focused on the individual. People who are committed to something take initiatives, make sure things happen—and that's the kind of people that we at any rate have had, and things are working very well as they are, I think. (Physician)	Committed PRs make sure things happen, and that works very well	Patient representatives’ participation in the group: a demanding role with different expectations from the professionals

### 2.5 Ethical considerations

The study followed the Code of Ethics of the World Medical Association (Declaration of Helsinki).^20^ Approval was obtained prior to the study from the local regional ethical review board (D.nr 2017/123). Participants were given written information about the study, including that participation was voluntary, the recorded interviews would be treated confidentially, and the participants could withdraw at any time without an explanation. Written informed consent was obtained from all individuals before starting the interviews.

## 3 RESULTS

**FIGURE 1:**
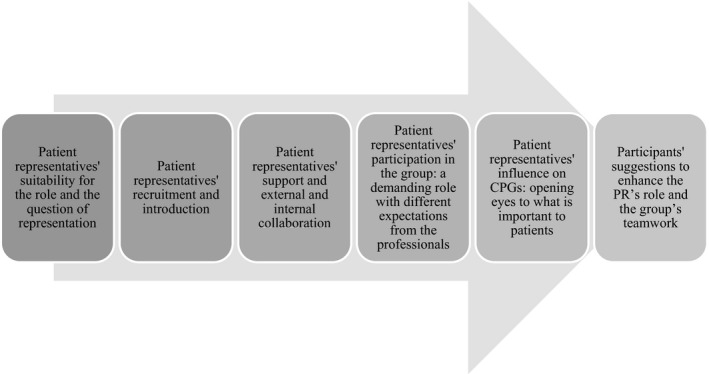
Overview of the categories

Professionals reported that PRs’ participation in the group opened their eyes to what patients viewed as important and that patients’ opinions were relevant to developing health care. However, several objections were also raised by both professionals and PRs. The six categories that emerged in the analysis (Figure 1) corresponding to various phases of PRs’ involvement in the groups ranging from recruitment through aspects of the work, to possibilities for improvement are described below, illustrated with quotations from PRs, RNs and physicians.

### 3.1 PRs’ suitability for the role and the question of representation

Professionals and one PR mentioned that PRs should have some distance from their illness and be in a stable phase of the disease before taking part in medical teamwork.

The important thing is that the patient is through with his or her treatment and is healthy or whatever or isn’t currently undergoing treatment. I’ve participated in other groups where there were patient representatives—and the patient has just gone through a treatment or hasn’t yet gone to the first follow‐up, for example—[and] that I feel is totally unethical, putting someone like that in a care program group. (RN)

Professionals reported PRs who used out‐of‐date information, presented their own experiences as general facts, did not fulfil their roles as members of the group, became annoyed when their opinions were neglected or viewed themselves as victims as disadvantages who affected the work negatively. A few physicians feared that more uninhibited PRs might ‘take over’ the group.

Physicians stated that uncertainty about PRs’ representativeness was the largest general problem with patient representation, and nurses said that PRs were not always able to be objective. Both professionals and PRs perceived a problem if PRs were not supported by a patient organization. PRs said there would be of advantage to be more than one PR in the group and believed that professionalizing the role would improve their representativeness. PRs also found it a challenge to decide whether their own opinions were the very best for all patients.

It’s not about a patient participating, but rather someone who can convey the patient perspective on the care. That’s one heck of a difference. The care side always says we focus entirely on the patient and it’s not relevant; but it’s more about bringing patients’ experiences into the decision‐making process in the care and in the planning, and that has to be taken more seriously. It’s not about the individual patient’s own experiences, but more about the necessity of securing broader support so that you represent patients collectively. (PR)

### 3.2 PRs’ recruitment and introduction

Recruiting and introducing PRs to the group and the work varied widely between CPGs. Some groups required recruitment through patient organizations, while others recruited through RCC patient education programmes, health‐care personnel or other PRs. Professionals reported difficulties in recruiting suitable PRs, the heavy workload could make the position unattractive.

Overall, it’s been difficult to find someone, and it has also been hard for them to participate. (Physician)

Having a bad prognosis, poor health condition or cognitive impairment from the disease or treatment could also affect a patient's ability to participate. PRs mentioned lack of time as an influencing factor, especially for people already quite busy or who had other responsibilities. Patients might also want to leave the disease behind, and younger patients could have difficulty identifying themselves with the majority of patients who were older. Both PRs and professionals suggested the option of recruiting patients’ relatives instead of patients or as a complement to add another dimension to the group's considerations. In some groups, no one formally introduced PRs; in others, the chairperson, another PR or the local RCC representative made the introductions.

### 3.3 PRs’ support and external and internal collaboration

PRs considered it their responsibility to prepare for meetings by reading all the information, even though they sometimes did not understand some of it. Only occasionally did PRs have someone in the group designated to help. PRs described sometimes feeling as if walls were built between different medical specialists during the meetings. This feeling was consistent with their experiences as patients in the health‐care system. They reported that each separate unit of health care often functioned well but also that collaboration between different units and clinics as well as within the administration needed improvement.

It’s not that the collaboration is poor, not at all. But they could be a little more open with each other, I mean, the different parts of the profession—the neurologists, the surgeons, the oncologists, and so on. Sometimes it feels like there are invisible walls separating them. (PR)

Support from a patient organization was appreciated, but it could also be the source of conflicting views and attitudes that made it difficult for PRs to contribute their best. A designated support person at an RCC could assist the PR in adopting a professional view. Support could also be helpful to PRs coping with issues discussed in the group, such as mortality and financial constraints. PRs who had received education through the RCC before their assignment to a group stated they would like a second course to refresh their knowledge.

### 3.4 PRs’ participation in the group: a demanding role with different expectations from the professionals

The professionals considered the PRs’ role important, but difficult and unclear, but their understanding and acceptance of the role had increased over time, especially in physicians. The initial introduction of the PRs to the group was important. If the first meetings were dismissive, PRs reported that their participation could be impaired.

Low expectations generally hampered PRs’ contributions, but attitudes towards PRs could depend on their background. PRs who had worked in health care or were academics said that their background could affect how positively they were addressed. Professionals mentioned that PRs’ participation could suffer from a lack of knowledge about medical issues and health‐care organization. Physicians generally listened more to PRs’ opinions if they could refer to studies, while RNs were more interested in their experiences as patients.

Yes, it’s when the discussion gets under way. That’s when, if you’re not keeping up, you can’t really do anything. They talk fast, with lots of abbreviations… lots of things are not described fully, things everyone knows. And you can’t ask them to adapt their way of talking just because there is a patient representative present. I don’t think you can ask that. They’re supposed to be doing their job, not explaining things. (PR)

PRs focussed on different areas than other group members depending on the topic. In line with many physicians’ expectations, PRs found it easier to engage in topics such as nursing care, availability of health care, patient–health professional interactions, rehabilitation and self‐care.

PRs believed their presence changed attitudes within their groups: members became more polite to each other, hierarchies faded, discussion of patient preferences increased, and verbal fights or insults between specialists decreased. However, professionals sometimes thought the presence of PRs obstructed their discussion of sensitive topics or was a nuisance when necessary explanations slowed discussions. Medical language could be difficult for PRs to understand, resulting in PRs feeling excluded, but it could also be difficult for professionals consulting from other specialties.

Because that’s something you might feel yourself. I might feel that when I’m in the large group with all the doctors, geneticists, statisticians, and so on—I might feel like “Here they go again, talking this incomprehensible jargon.” I actually haven’t the faintest idea what they’re talking about. And if you want to try and speak up, you feel so small in that context, and I imagine that’s how it must feel for the patient representative, too, in that little group. (RN)

Professionals sometimes protected the PRs from sensitive topics such as discouraging prognoses. Nurses wanted PRs to be invited to participate in all discussions, but also be allowed to absent themselves if they did not want to discuss delicate matters. Some professionals found some topics uncomfortable to discuss in the presence of PRs and wanted meetings without PRs to discuss those matters more openly.

PRs wanted to find solutions that suited both patients and health‐care providers, rather than to complain about what was not perfect. Both PRs and professionals found the teamwork interesting and rewarding. Nurses found it educational when patients’ perspectives differed from professionals’ assumptions and argued that because most group members were physicians, at least two PRs should be included in each group. They also suggested that patients’ relatives made excellent participants who could report an entirely different description of the disease. Professionals regarded PR contributions as beneficial to patients in the short term and necessary to CPG development in the long term.

### 3.5 PRs’ influence on CPGs: opening eyes to what is important to patients

PRs asserted that only patients could communicate the true patient perspective: they could describe how it felt to lose physical or mental functions to disease or treatment. They could focus on ‘what's in it for the patient?’ when new methods or treatments were suggested. PRs asked to focus on existential questions and pointed out that they also have useful experiences of society beyond the world of health care.

Professionals’ expectations varied widely. Some had no expectations at all of the PRs, others expected them to comment on certain issues or to represent the group to patient organizations. Sometimes text was marked before meetings to guide PRs’ comments or time was allocated during meetings for PRs’ opinions. In some groups, PRs were fully responsible for writing certain parts of the CPG.

PRs made an effort not to compete for dominance, but they wanted to have an active role, even when expectations were low. This required both courage and knowledge as the rest of the team did not always welcome their taking an active role. Although PRs could sometimes influence changes to a CPG (although seldom in medical areas), they tended to give in rather easily during discussions. Although they were convinced of incongruences between different parts of the CPG, they found it challenging to persuade the professionals to read each other's parts.

Some physicians thought that PRs had influenced the CPG towards a patient's perspective, while others declared that PRs had had no influence at all.

Yes, [they] definitely affected…. I entirely agree that they have opinions that we listen to and take into consideration. When it comes to patient information sheets, how you write things and so on, they are most definitely involved and have considerable influence. (Physician)

No, I’ve thought about that and I cannot give you any example of it. He’s put forth a number of views, but he hasn’t said anything that we’ve ultimately taken into consideration. (Physician)

Nurses described that the CPGs were based on discussions within the group, with no one superior to the others.

### 3.6 Participants’ suggestions to enhance the PR’s role and the group's teamwork

Both PRs and professionals wanted written definitions of the PR role, including terms, expectations and agreements with appropriate patient organizations on sharing information. They also underlined the importance of clarifying that the PR is not just a participating patient, but contributor best placed to communicate the patient perspective.

Professionals mentioned educating PRs not only of their role in HCWGs, but also of basic knowledge of medicine and the structure of health care. Both PRs and professionals expressed the importance of continuous support and guidance. Several participants suggested that having more than one PR in the group could make them less fragile, strengthen their role and give them peers to talk to. PRs also thought that forums for PRs to meet might make them feel more confident. Both PRs and professionals underlined the importance of an open approach in meetings, allowing all members to participate in discussions and expecting everyone to explain their positions using common language understandable to all.

PRs suggested using simultaneous digitalized writing and video meetings to gain flexibility and reduce travel time. They also suggested that increased economic support could give them more time to prepare for meetings and engage with patient organizations and networks.

A little compensation for the time we put in, nothing more. There should be more, so you can have two days of compensation for a one‐day meeting, so you have more time to prepare and to communicate with other patients and so on. That would make it better for the patient. (PR)

## 4 DISCUSSION AND CONCLUSION

### 4.1 Discussion

Participants in this study mostly considered the PRs’ role important, but also raised several objections. PRs’ contributions were described as often disregarded, hampered by the uncertainty of the role and the low expectations of other group members. Some professionals also questioned the PRs’ representativeness or said they were unwilling to discuss all topics in their presence.

In several international settings, patient participation in health‐service development is a mandatory part of health‐care policy^21^, ^22^ implemented on all levels from direct care to society and government.^7^, ^8^, ^9^ Although the Swedish *Patient´s Law* has strengthened, clarified and promoted the position of patients and confirmed their integrity, self‐determination and participation,^23^ the present results show that professionals still have low expectations and a lack of respect for PRs. PRs are invited to participate in the working groups and spend time and effort on the work, but professionals do not fully utilize their engagement and knowledge.

PRs described their role in the CPG‐monitoring groups as demanding and sometimes unclear, while professionals in the groups generally reported a lack of information about the role of PRs. The issues of role description and expectations of PRs, raised in several previous studies and reviews,^1^, ^4^, ^11^, ^12^, ^17^, ^24^, ^25^ still seem to be key factors in facilitating or creating barriers to PPI. A succinct description of the role would benefit both PRs and professionals in HCWGs. A 2017 systematic review showed that despite recommending the involvement of PRs, very few guidance documents provided any structure or guidance on how to do this.^26^ To clarify the role and goals of PRs in CPG‐monitoring groups, both PRs and patient organizations for the affected disease groups need to be involved.

While physicians numerically dominated the teams and preferred the medical view of the disease, the nurses and PRs, as minorities in the groups, engaged more easily on topics not strictly medical, but still very important for patient care. PRs pointed out that their participation in medical discussions might be more important as patients’ experiences could otherwise be disregarded in these areas. However, medical language can be a barrier and PRs often feel isolated during meetings.^4^, ^12^, ^24^, ^27^, ^28^ In this study, when PRs did not always understand the medical terminology, they felt inferior or excluded. Explanations could be helpful, but physicians noted that they took time and slowed group meetings. When the language was adjusted, however, PRs could understand, be involved and affect discussions with their perspectives. Adjusting language and explaining complex issues could also benefit other group members, as even professionals shared in the interviews that they did not understand everything discussed in the groups. These findings support the need to provide PRs with assistance on complex scientific and technical issues to optimize their participation.

Teamwork is necessary for organizational success.^14^ It is evident that working groups with permanent members, as in this study, could reward the efforts of team development, but only if certain conditions are met and commitment is required.^14^ An interesting finding was that when laypersons were involved in the teamwork, the attitudes between members in the group improved over time. Working with service users in groups has been shown to change health professionals’ attitudes, values, their beliefs about the value of users’ involvement, and their attitudes towards other professionals, although such teamwork is still described as a difficult task.^2^ Considering these other findings, it might have been interesting for us to ask the professionals how long they had been attached to the working groups, but our focus was on the PRs’ experiences over time.

Excluding PRs when discussing sensitive questions such as bad prognoses or limited treatment options, as suggested by some professionals, could impair the teamwork. The professionals were concerned about the PRs’ feelings, but none of the PRs mentioned having any problem discussing such topics. Evidence of PPI’s impact on CPG underscores the importance of engaging PRs, and CPG without PPI input could actually be challenged as invalid.^12^ The most important point could be whether the group views the PR as a valuable member or a barely noticed symbol. Patient engagement can become tokenistic,^1^, ^29^ as expressed by some professionals in this study. Changing attitudes and roles have been reported as necessary to developing trust, and translating traditional, scientific, and clinical vocabulary could help to reduce PRs’ (and other professionals’) feelings of exclusion.^30^

This study shows that PRs add important knowledge and have an impact on CPGs. However, expectations of PRs differ widely and their inclusion in CPG‐monitoring groups has been reported to be only partly successful.^28^ PRs should not only be invited to meet with the group, but also to take part in discussions based on respect, a key characteristic of patient participation.^5^ PRs in this study felt that they had affected the CPGs more than the professionals, especially the physicians, acknowledged. That may reflect a difference in priorities between physicians, who have a stricter focus on medical issues, and PRs and nurses, whose foci are broader. PRs in this study agreed with the findings that some issues could be overlooked by professionals and that PRs could help to identify patient‐relevant topics and outcomes, as previously described.^12^

The representativeness of the PRs was an important question, especially for physicians, and has been presented as a barrier.^4^ This doubt may also help to explain some professionals’ resistance to patient participation.^1^, ^4^, ^24^, ^28^ Some group members described it as a problem if the PRs’ opinions were not supported by a patient organization, but others were concerned that including only members from patient organizations risked missing different important views. Whether a PR should be expected to represent the views of an organization or to deliver their own personal experience remains a key recruitment question.^13^ Work descriptions used by some HCWGs may assist in clarifying expectations.^13^

Support for PRs seems an essential area for improvement. Support and training, the two most frequently reported facilitators in a review,^4^ have also been identified as key conditions for meaningfully involving PRs.^13^ Being objective and expressing personal opinions are demanding tasks for any PR. PRs in this study suggested involving a group of patients rather than only one, as has previously been described to facilitate PPI in establishing guidelines.^4^ Some PRs suggested professionalizing the PR role and defining its responsibilities and necessary knowledge.

As early as 1978, the World Health Organization stated that ‘people have the right and duty to participate individually and collectively in the planning and implementation of their health care’.^22^ Much work has been done since then, but our results show that more is needed. Health‐care professionals need to embrace what patients themselves describe as participation to create the best conditions for that participation.^5^ Patients’ descriptions focussed on interacting with health professionals, rather than merely taking part in decisions, and on having knowledge rather than being informed.^5^ All group members need to work together to achieve those goals. If PRs were considered equal team members and respected and resourceful individuals^5^ and given adequate resources, including cultural and financial,^31^ then patient participation could be realizable and influence the prioritization of patient‐relevant topics^11^, ^12^ and the implementation and dissemination of CPGs.^12^

One strength of this study is the inclusion of participants with different professions and roles from seven CPG monitoring groups for cancer from different health‐care regions across Sweden. This approach captures various experiences, prevents the influence of the culture of one particular group, and increases the transferability of the findings to other groups.^32^ The PRs interviewed were all permanent members of the groups, but had been involved for various periods of time, so both immediate and profound reflections were captured. To achieve trustworthiness, all authors were involved in all steps of the research process including interviews, analysis and writing up the qualitative findings.^32^ Nevertheless, influences of pre‐understandings and prejudices could have biased the results. The authors’ close collaboration, however, with repeated face‐to‐face discussions of the content and coding of the interviews, establish that the findings are derived from the data.

One limitation is that all interviews, but one, were conducted by telephone instead of face‐to‐face, but this approach did allow us access to a nationwide group of participants. The PRs’ suitability for the role was mainly mentioned by the professionals; only one PR expressed such concerns. This was not put as a direct question to the participants, which may be considered a mistake. However, most fears expressed by the professionals were never reported as actually experienced.

Another limitation is the inclusion of PRs from working groups focussed only on cancer. However, most issues of concern for cancer patients would probably be recognized by other patients, although perhaps from different angles or to a lesser degree. Eventually, the PRs were only information givers. It would have been valuable if PRs also had been part of the research process.

### 4.2 Conclusion

This study highlights the fundamental problems that remain to be solved despite the long involvement of PRs in HCWGs. Even though the PR role and teamwork differed between the groups, most PRs need to be empowered to be actively involved in the teamwork and have their engagement and knowledge fully utilized. Clarifying the roles of all participants and their shared goals is necessary to establish an effective working group. Enhancing teamwork through clarifying roles and expectations could lead to more inclusive and equal teams able to work more effectively towards the goal of improving health care.

### 4.3 Practice implications

These findings could help HCWGs collaborate with PRs to develop their role. This knowledge could support working groups in improving systems for selecting, introducing and supporting PRs, as well as offer tools for ongoing evaluations and improvements of the teamwork.

## CONFLICT OF INTEREST

The authors declare no conflicts of interest.

## Data Availability

We regret that there will be some difficulties to share the data utilized in the article. The transcribed interviews could be requested from the corresponding author, but the interviews are unfortunately only presented in Swedish. Also, there would be concerns due to ethical restrictions as the informants’ privacy is granted by the local Regional Ethical Review Board in Sweden. However, all authors declare that all data were collected as described in the Material and Methods sections and that the data sets, including citations, are completely and correctly reproduced.
